# Role of A_2B_ adenosine receptor-dependent adenosine signaling in multi-walled carbon nanotube-triggered lung fibrosis in mice

**DOI:** 10.1186/s12951-019-0478-y

**Published:** 2019-03-29

**Authors:** Biying Liu, Qizheng Bing, Siyu Li, Bing Han, Jingjing Lu, Ruiqi Baiyun, Xiaoya Zhang, Yueying Lv, Hao Wu, Zhigang Zhang

**Affiliations:** 1 College of Veterinary Medicine, Northeast Agricultural University, 600 Changjiang Road, Harbin, 150030 China; 2 Heilongjiang Key Laboratory for Laboratory Animals and Comparative Medicine, 600 Changjiang Road, Harbin, 150030 China

**Keywords:** Multi-walled carbon nanotubes, Lung fibrosis, Adenosine, A_2B_ adenosine receptor, Transforming growth factor-β1, Follistatin-like 1, Fibroblast-to-myofibroblast transition, High-performance liquid chromatography

## Abstract

**Background:**

Multi-walled carbon nanotube (MWCNT)-induced lung fibrosis leads to health concerns in human. However, the mechanisms underlying fibrosis pathogenesis remains unclear. The adenosine (ADO) is produced in response to injury and serves a detrimental role in lung fibrosis. In this study, we aimed to explore the ADO signaling in the progression of lung fibrosis induced by MWCNT.

**Results:**

MWCNT exposure markedly increased A_2B_ adenosine receptor (A_2B_AR) expression in the lungs and ADO level in bronchoalveolar lavage fluid, combined with elevation of blood neutrophils, collagen fiber deposition, and activation of myeloperoxidase (MPO) activity in the lungs. Furthermore, MWCNT exposure elicited an activation of transforming growth factor (TGF)-β1 and follistatin-like 1 (Fstl1), leading to fibroblasts recruitment and differentiation into myofibroblasts in the lungs in an A_2B_AR-dependent manner. Conversely, treatment of the selective A_2B_AR antagonist CVT-6883 exhibited a significant reduction in levels of fibrosis mediators and efficiently decreased cytotoxicity and inflammatory in MWCNT treated mice.

**Conclusion:**

Our results reveal that accumulation of extracellular ADO promotes the process of the fibroblast-to-myofibroblast transition via A_2B_AR/TGF-β1/Fstl1 signaling in MWCNT-induced lung fibrosis.

## Background

Carbon nanotubes (CNTs) are new nanomaterials in a single layer (single-walled CNT, SWCNT) or concentric multi-layers (multi-walled CNT, MWCNT) with increasingly wide utilization in the fields of medicine, electronics, and structural engineering [1]. However, because of the high demand for CNTs, it must be concerned that the health hazards of CNTs for human occupational and environmental exposure are more serious [2, 3]. One study reported MWCNT-containing airborne dust levels up to 400 μg/m^3^ in a research laboratory, although the total mass concentration reported in this study was not comprised exclusively of MWCNT [4]. Furthermore, some animal studies have confirmed that pulmonary exposure to MWCNT results in fibrosis in the lungs [5–7].

Once exposure, MWCNTs deposit in the respiratory tract and increase lung burden, eventually leading to chronic inflammation and high risks of related adverse effects such as fibrosis [8]. The pathologic development and features of CNT-induced pulmonary interstitial fibrosis overlap with those of irreversible pulmonary fibrosis (IPF) and pneumoconiosis considerably. IPF is the main cause of death due to unknown pathogeny and few treatment options. The mortality rate of IPF at 3–5 years after diagnosis is 50% [9]. The fibrosis is progressive post-exposure and characterized by fibroblast proliferation and an excessive deposition of extracellular matrix (ECM) in the interstitium. Fibroblasts in granulation tissue differentiate into myofibroblasts, which has contractile phenotype and proliferates and synthesizes ECM components [10].

Secretion of inflammatory cytokines and infiltration of additional inflammatory leukocytes were observed after pulmonary exposure to MWCNT, which confirms the pulmonary inflammation produced by MWCNT [6, 11]. Elevated population of neutrophils is correlated with the acute inflammatory response [12]. Myeloperoxidase (MPO) is a peroxidase enzyme that is synthesized and secreted by neutrophils and monocytes [13]. MWCNT deposit efficiently and persistent in the respiratory tract, due to their pro-inflammatory potency represent one environmental factor proceeding to the progressive fibrotic lesions [14]. There have been significant advances in understanding of MWCNT toxicity, yet the underlying mechanism of MWCNT-induced lung fibrosis remains elusive. High levels of extracellular adenosine (ADO) were produced in response to tissue injury and inflammation [15]. Moreover, extracellular ADO levels are closely associated with the progression and severity of pulmonary fibrosis [16, 17]. Therefore, the aim of the present study is to determine the ability of ADO to elicit lung inflammation and exacerbate lung fibrosis in MWCNT treated mice.

In response to cellular stress, adenosine triphosphate (ATP) is released into extracellular and subsequently dephosphorylated to ADO by ecto-nucleotidases including ectonucleoside triphosphate diphosphohydrolase 1 (CD39) and Ecto-5′-nucleotidase (CD73) [18]. ADO plays a principal role in the wound healing process. Under physiologic conditions, extracellular ADO level in cells and tissue fluids are in the nanomolar range, while ADO rises substantially during different forms of cellular distress [19]. ADO orchestrates the cellular response by acting on ADO receptors, including A_1_ adenosine receptor (A_1_AR), A_2A_AR, A_2B_AR, and A_3_AR, of which A_2B_AR has emerged as a major mediator of chronic lung disease, such as fibrosis and tissue remodeling [20]. A_2B_AR has the lowest affinity for ADO and is normally activated under excess accumulation of extracellular ADO. A_2B_AR levels are elevated in patients with IPF [21]. The ADO signaling system has been closely linked with the production of several mediators, including interleukin-6 (IL-6) [6] and transforming growth factor (TGF)-β1 [22]. In addition, ADO contributes to the differentiation of pulmonary fibroblasts into myofibroblasts, disease progression, and tissue remodeling via the engagement of the A_2B_AR [23]. However, the ADO signaling involved in MWCNT-induced lung fibrosis is unknown until today.

TGF-β1 is a profibrotic cytokine that promotes myofibroblast activation and proliferation and plays a central role in the induction of fibrogenesis [24]. Exaggerated TGF-β1 signaling contributes to accumulation of collagen and further ECM [25]. Interestingly, the inhibition of A_2B_AR exerts an antifibrotic effect in chronic lung disease by preventing the expression of TGF-β1 [26]. Therefore, there is a necessity to further characterize the A_2B_AR interacting with TGF-β1 signaling in MWCNT-induced lung fibrosis.

The hypothesis in this study is that the progressive inflammation, alveolar remodeling, and lung fibrosis induced by MWCNT are associated with progressive and proportionate increases in the level of extracellular ADO and the enhanced expression of A_2B_AR. Moreover, we speculate that CVT-6883 (a selective A_2B_AR antagonist) would reduce TGF-β1-mediated fibroblast proliferation and differentiation into myofibroblasts, and ultimately attenuate MWCNT-induced lung fibrosis.

## Results

### MWCNT characterization

Physicochemical characteristics and morphology of the MWCNT sample used in the present study are reported in Table 1. The surface morphology of MWCNTs is shown in micrograph images.

**Table 1:** Physicochemical properties of MWCNT samples

Sample	Diameter	Length	Morphology^b^
MWCNT	30–50 nm^a^	< 10 μm^a^	

^a^Refer to Chengdu Organic Chemistry Co., Ltd (http://www.timesnano.com)

^b^Scanning electron microscope for TNM7

### MWCNT-induced fibrotic phenotypes in mice

**Fig. 1:**
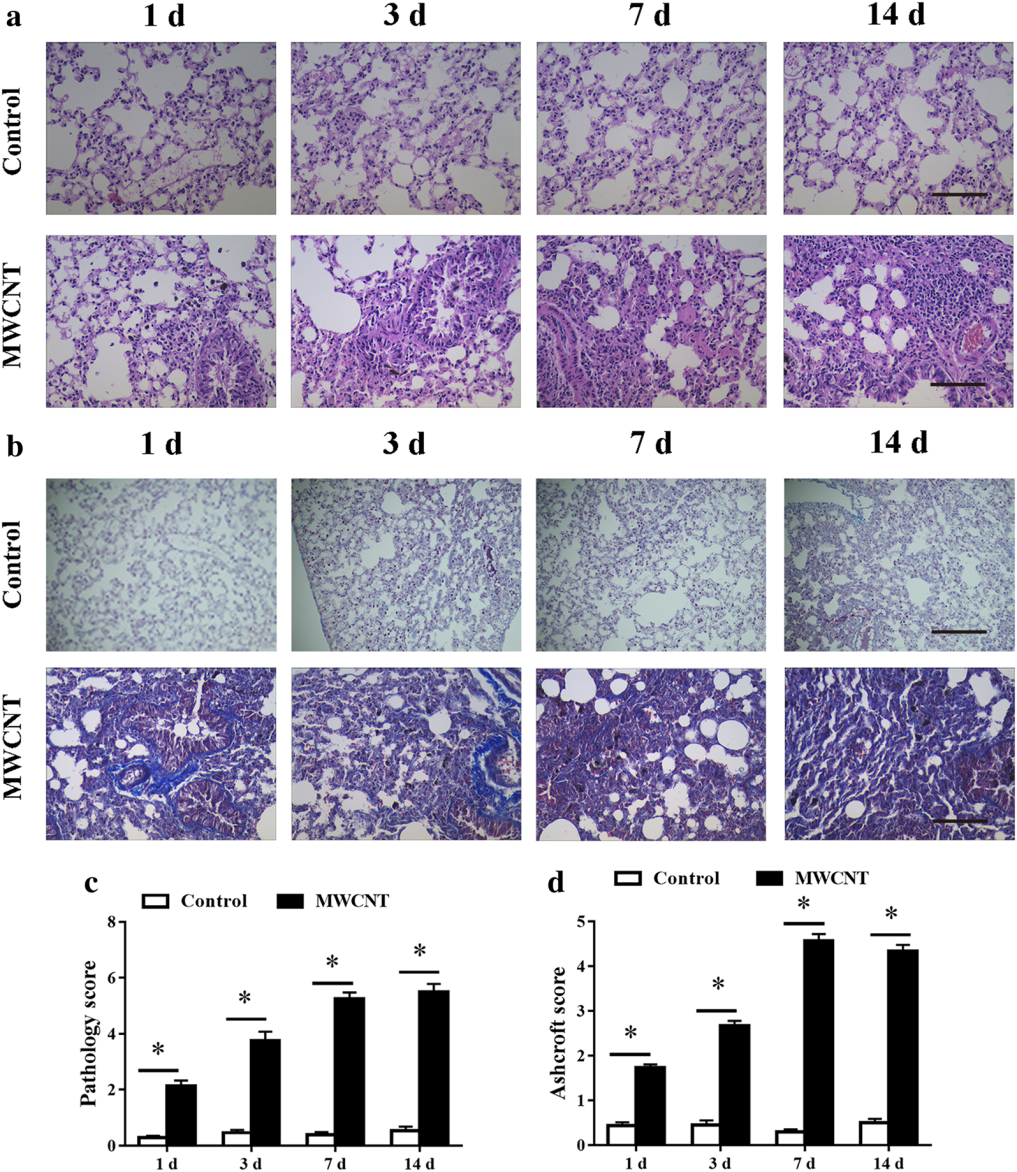
MWCNT induced lung injury and fibrosis in mice. Mice received DM (50 μL) or MWCNT (40 μg/50 μL DM) and were killed on days 1, 3, 7, and 14 post-exposure. **a** Pathological effects of lung tissues were evaluated by H&E staining (×200), bars = 20 μm. **b** Collagen fbers were detected in blue color by Masson’s trichrome staining (×200), bars = 20 μm. **c** Lung pathology score in H&E stained samples. **d** Quantification of lung fibrosis on histological specimens was performed using a numerical scale. Data represented the mean ± SEM (n = 6). *p < 0.05, versus control group

The histopathological changes in the MWCNT and DM groups have been shown in Fig. 1. Lung tissues were obtained at 1, 3, 7, and 14 days post-exposure. H&E staining showed interstitial thickening and bronchiolocentric inflammation in the mice exposure to MWCNT. The pathological changes reached a peak on day 7 and persisted throughout the 14-day post-exposure. As expected, aspiration of DM did not cause notable changes in the lungs (Fig. 1a).

We performed Masson’s trichrome staining to examine the fibrotic response directly. Abnormal collagen deposition was observed in the lung of MWCNT treatment on day 1 post exposure, progressed to a peak level on day 7, and maintained at a similar level throughout the day 14 (Fig. 1b). However, fibrotic mass formation was not observed in mice treated with DM.

MWCNTs exposure led to a significant increase in disease pathology compare to DM control (Fig. 1c). The fibrotic changes were quantified by using the Ashcroft score, which confirmed a significant increase of the fibrotic lesions in MWCNT treatment lungs compared with control at all the time points examined (Fig. 1d).

### The effect of MWCNT on CD73, ADO, and A_2B_AR

**Fig. 2:**
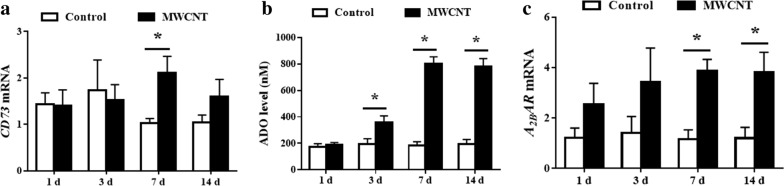
The mRNA expression of CD73 and A_2B_AR in lung tissues, as well as ADO level in BALF at 1, 3, 7, and 14 days after MWCNT and DM treatment. **a** The transcript expression of CD73. **b** ADO levels. **c** The transcript expression of A_2B_AR. Data represented the mean ± SEM (n = 3). *p < 0.05, versus control group

Treatment of mice with MWCNT significantly increased CD73 gene expression on whole lung homogenate at 7 days post-exposure. However, CD73 gene expression appeared to be more variable at day 14 post exposure (Fig. 2a).

We measured the level of extracellular ADO, as defined by relative ADO level in bronchoalveolar lavage fluid (BALF). As expected, MWCNT treatment significantly elicited ADO level in BALF from day 3 to 14 (Fig. 2b).

MWCNT exposed mice exhibited significantly increased in the mRNA expression of A_2B_AR at 7 days post-exposure, and this elevation remained throughout 14 days after MWCNT exposure (Fig. 2c).

### Inhibition of pulmonary inflammatory following treatment with CVT-6883

**Fig. 3:**
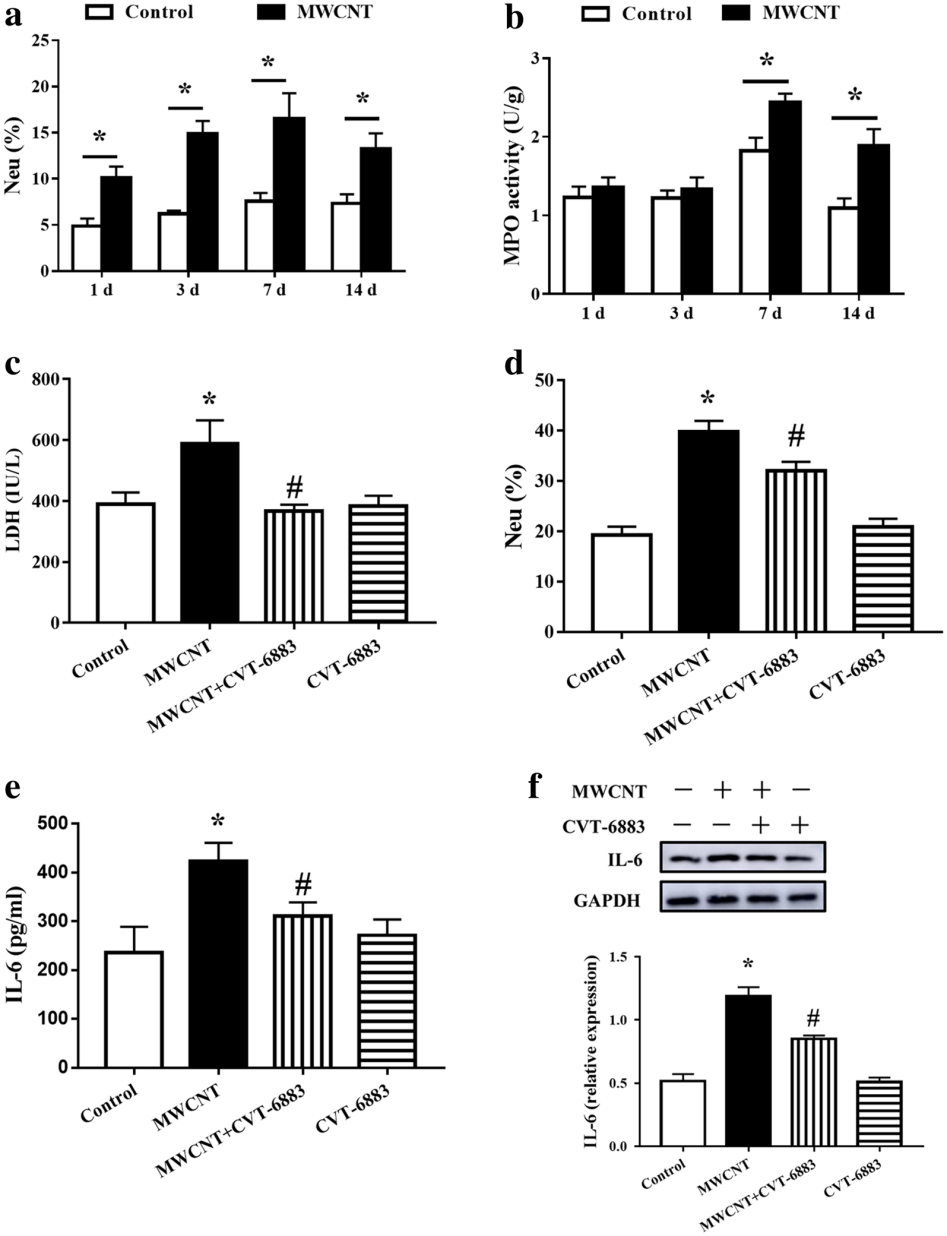
CVT-6883 attenuated MWCNT-induced lung injury in mice. **a** Percentage of neutrophils in peripheral blood and **b** MPO activity in the lung tissues were analyzed on days 1, 3, 7, and 14 post-exposure to a single dose of DM or MWCNT (mean ± SEM, n = 6). **c** LDH activity in serum and **d** percentage of neutrophils in peripheral blood were analyzed on day 7 post-exposure (mean ± SEM, n = 6). **e** IL-6 in BALF from mice on day 7 post-exposure was determined using ELISAs (mean ± SEM, n = 3). **f** Western blot analysis of IL-6 in lung tissues. *p < 0.05, versus control group. ^#^p < 0.05, versus MWCNT group

To evaluate MWCNT-induced inflammatory, we examined percentage of neutrophils in peripheral blood (Fig. 3a) and MPO activity (Fig. 3b) in the lung of mice. MWCNT induced a rapid increase in the percentage of neutrophils, and continued to increase on day 7 followed by a slightly reduction on day 14. MWCNT treatment enhanced MPO activity from day 7 to 14.

Based on this time course of fibrosis development induced by MWCNT, day 7 was chosen as the time point to reflect the MWCNT-induced lung injury. MWCNT exposure significantly increased lactate dehydrogenase (LDH) activity in serum (Fig. 3c) and percentage of neutrophils (Fig. 3d) in peripheral blood. Treatment with CVT-6883 caused a noticeable alleviation in LDH and percentage of neutrophils produced by MWCNT.

We further examined the protein levels of IL-6 in BALF (Fig. 3e) and lung tissues (Fig. 3f). The levels of IL-6 significantly increased in BALF and lung tissues of the MWCNT-induced group as compared with control mice. In contrast, this augmentation was significantly inhibited by the treatment of CVT-6883.

### Treatment with CVT-6883 reduced profibrotic mediators in the lungs

**Fig. 4:**
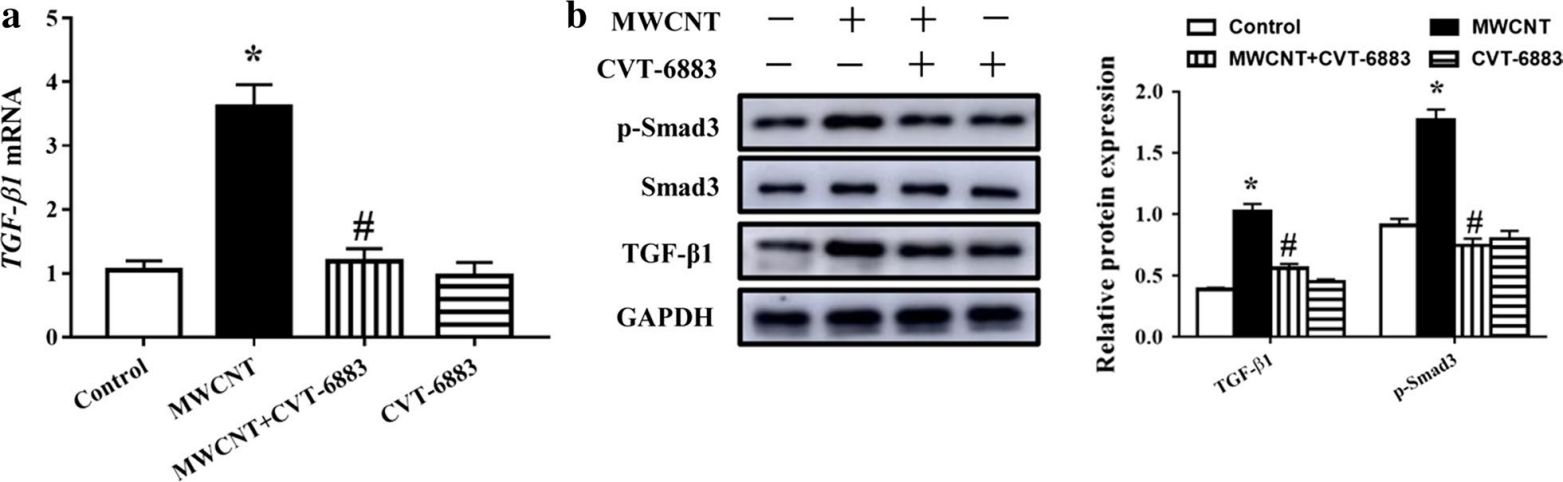
CVT-6883 inhibited profibrotic mediators in MWCNT exposure mice. **a** RT-qPCR was performed to determine the relative mRNA level of TGF-β1 in lung tissues. **b** The protein levels of TGF-β1, p-Smad3, and Smad3 in lung tissues were determined using western blot. Data represented the mean ± SEM (n = 3). *p < 0.05, versus control group. ^#^p < 0.05, versus MWCNT group

MWCNT treatment resulted in a significant rise in TGF-β1 mRNA and protein expression. Moreover, our data showed that MWCNT treatment significantly promoted TGF-β1-stimulated Smad3 phosphorylation (p-Smad3) in lung tissues. Treatment of CVT-6883 showed a significant decline in TGF-β1 mRNA expression (Fig. 4a) and TGF-β1 and p-Smad3 protein levels (Fig. 4b).

### Normalization of lung fibrosis in the lungs of CVT-6883-treated mice

**Fig. 5:**
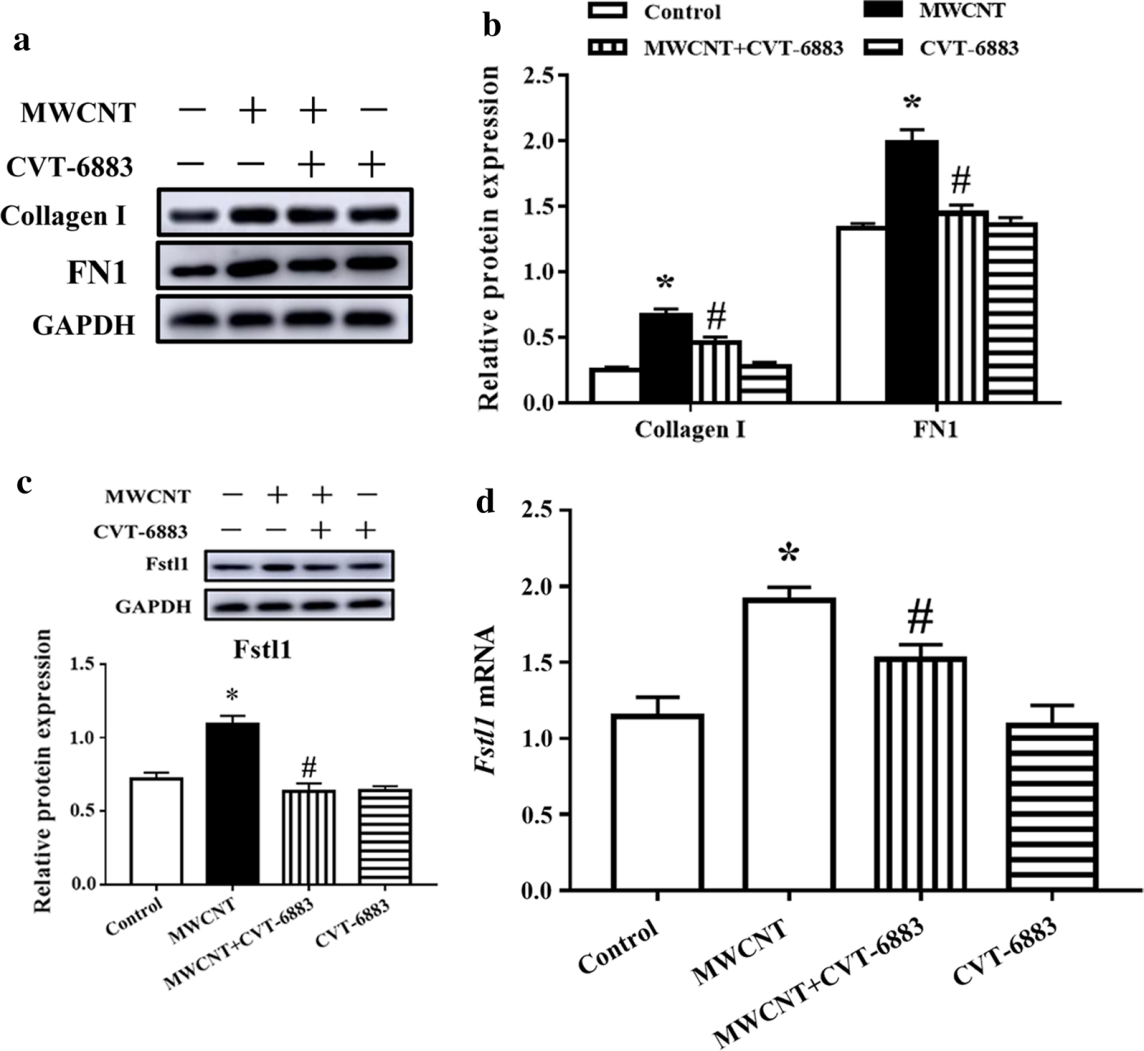
CVT-6883 decreased accumulation of fibrotic matrix protein and inhibited activation of Fstl1 in MWCNT exposure mice. **a** The protein levels of collagen I and FN1 were determined by immunoblotting. Quantified protein levels are shown in (**b**). **c** The protein levels of Fstl1 were determined by immunoblotting. **d** RT-qPCR was performed for Fstl1. Data represented the mean ± SEM (n = 3). *p < 0.05, versus control group. ^#^p < 0.05, versus MWCNT group

To further determine the effect of A_2B_AR in the lung fibrosis of MWCNT exposure, we examined the protein level of two major ECM proteins (collagen I and fibronectin 1 (FN1)). As expected, CVT-6883 treatment inhibited the increased in collagen I and FN1 levels induced by MWCNT (Fig. 5a, b).

### CVT-6883 inhibited fibroblast-to-myofibroblast transformation in mouse lungs

**Fig. 6:**
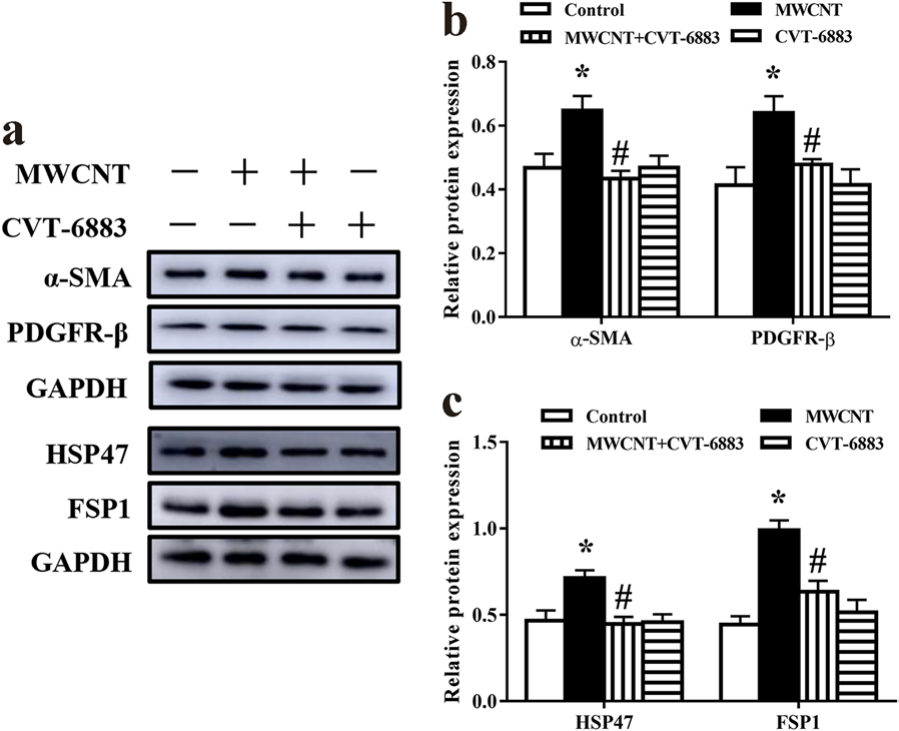
CVT-6883 inhibited fibroblast-to-myofibroblast transformation in MWCNT exposure mice. **a** Immunoblotting of myofibroblast marker α-SMA and PDGFR-β, fibroblast marker HSP47 and FSP1. Quantified protein levels are shown in (**b**, **c**). Data represented the mean ± SEM (n = 3). ^*^p < 0.05, versus control group. ^#^p < 0.05, versus MWCNT group

To determine whether A_2B_AR have the potential to directly induce fibrotic reactions characterized by increased differentiation of fibroblasts into myofibroblasts, we analyzed the effect of CVT-6883 on the expression of follistatin-like 1 (Fstl1) and fibroblast-to-myofibroblast transition markers: α-smooth muscle actin (α-SMA), platelet-derived growth factor receptor-β (PDGFR-β), heat shock protein 47 (HSP47), and fibroblast-specific protein 1 (FSP1). Fstl1 has the regulatory functions in cell proliferation and differentiation. As shown in Fig. 5c and d, MWCNT enhanced mRNA expression and protein level of Fstl1, while CVT-6883 inhibited Fstl1 expression. In addition, the protein levels of α-SMA, PDGFR-β, HSP47, and FSP1 were dramatically increased by MWCNT in the lungs (Fig. 6). However, CVT-6883 cotreatment suppressed the effects of MWCNT.

## Discussion

CNT exposure induced the pulmonary collagen deposition accompanied with pronounced acute inflammation preceding chronic fibrosis progression. Serum LDH activities reflects the cellular injury after MWCNT exposure. To further evaluate potential pulmonary inflammation induced by MWCNT, the level of proinflammatory cytokines IL-6 and MPO activity between the BALF and lung tissues was examined. In our study, MWCNT induced pulmonary inflammatory response by recruiting and activating neutrophils in the lungs through movement of circulating leukocytes to the lungs. Previous studies have shown that MWCNT-induced inflammation is probably due to phagolysosome membrane permeability, which has been implicated in the activation of IL-6 [27]. Notably, CVT-6883, as an inhibitor of A_2B_AR, greatly ameliorated pulmonary inflammation, resulting in a significant reduction in fibrosis. Based on these results, collagen deposition caused by MWCNTs is related to the proinflammatory effects of MWCNTs. Notably, the blockade of A_2B_AR restores cellular injury and inflammatory effects, thereby alleviating the progression of lung fibrosis.

We firstly examined the expression of CD73 following MWCNT exposure. Interestingly, MWCNT-induced lung fibrosis significantly increased CD73 gene expression and ADO levels. This finding is consistent with CD73-mediated enzymatic conversion of adenosine monophosphate (AMP) to ADO in the lungs of mice with radiation-induced pulmonary fibrosis [28]. In addition, ADO also increased CD73 through transcriptional regulation via cyclic AMP response element in the CD73 promoter [28]. Chronic inflammation was associated with a constant increase in CD73^+^ leukocytes in the lung and an accumulation of CD73^+^ T cells, during the fibrotic phase [28]. CD73 was up-regulated in lung biopsy samples from patients with stage 4 chronic obstructive pulmonary disease or severe idiopathic pulmonary fibrosis, respectively [29]. Moreover, reduced extracellular ADO accumulation in radiation treated CD73^−^/^−^ mice prevented fibrosis development [30]. Thus, the necessity of ADO production of nucleoside signaling can be confirmed, and CD73 activation and ADO accumulation potentiates lung fibrosis after MWCNT treatment.

In the present study, elevations in extracellular ADO activated A_2B_AR, which promoted MWCNT-induced lung fibrosis through activating TGF-β1. Elevated expression of A_2B_AR has been detected in several chronic lung diseases, such as chronic obstructive pulmonary disease and lung fibrosis [21, 23]. The role of A_2B_AR stimulation or blockade in cell proliferation has been depending on the cell type and culture condition [31, 32]. Of note, A_2B_AR stimulated TGF-β synthesis in lung fibroblast cells [33]. Furthermore, A_2B_-null mice exhibit slightly effects in acute lung injury but reduced lung fibrosis, suggesting that A_2B_AR promote fibrosis [34]. We therefore determined whether A_2B_AR modulates TGF-β1 signaling in CNT-induced lung fibrosis. The TGF-β1/Smads signaling pathway occurs as a result of receptor-ligand interactions resulting in the expression of a number of TGF-β1 target genes through the rapid phosphorylation and nuclear translocation of Smad3 [35]. Inhibition of TGF-β/Smad signaling pathway is an effective approach to treat fibrotic disorders [36]. Our results showed that MWCNT remarkably stimulated the expression of TGF-β1 and Smad3 phosphorylation in the lungs in an A_2B_AR-dependent manner. These data identified the A_2B_AR as an upstream modulator of TGF-β1 and can be a potential therapeutic target in MWCNT induced lung fibrosis.

Large amounts of ECM remodels connective tissue into dense scar tissue, and ultimately leads to a disruption of organ architecture and loss of function [37]. TGF-β1 plays a central role in the ECM proteins production (such as collagen I and FN1) in the lungs after MWCNT exposure [10, 38]. Inhibition of TGF-β1 signaling by Smad3 inactivation was partially resistance to pulmonary fibrosis [39]. The results of present study are also consistent with this because MWCNT increased the production of collagen I and FN1 where TGF-β1 signaling was activated. Moreover, our study showed that induction of collagen I and FN1 was blocked by co-treating with A_2B_AR inhibitor CVT-6883, confirming the critical role of A_2B_AR in MWCNT-induced deposition of fibrous ECM. Altogether, the antagonism of A_2B_AR potentially suppresses the TGF-β1 signaling activation and thereby inhibits ECM production and deposition during MWCNT-induced lung fibrosis.

Lung fibrosis is characterized by excessive accumulation of α-SMA-expressing myofibroblasts arising from interactions with TGF-β1 and mechanical influences [40]. Fibroblasts/myofibroblasts are major effector cells in production of ECM proteins and airway remodeling [41]. Fstl1 is associated with myofibroblast accumulation and subsequently ECM production that is mediated by canonical TGF-β signaling [42, 43]. Previous study showed that MWCNTs increased the myofibroblast population by promoting fibroblasts proliferation and differentiation [44]. Here, blocking A_2B_AR signaling using CVT-6883 markedly attenuated Fstl1 induction in MWCNT-treated lung tissue. We further examined the effects of A_2B_AR in fibroblasts and myofibroblasts during MWCNT-exposed lungs using HSP47 and FSP1 as markers for fibroblasts and, α-SMA and PDGFR-β for myofibroblasts [44–46]. Our study found that MWCNT remarkably increased numbers of fibroblasts and myofibroblasts in the lungs in an A_2B_AR-dependent manner. Collectively, MWCNT elevates A_2B_AR expression, which promotes Fstl1-induced fibroblasts proliferation and differentiation.

## Conclusion

**Fig. 7:**
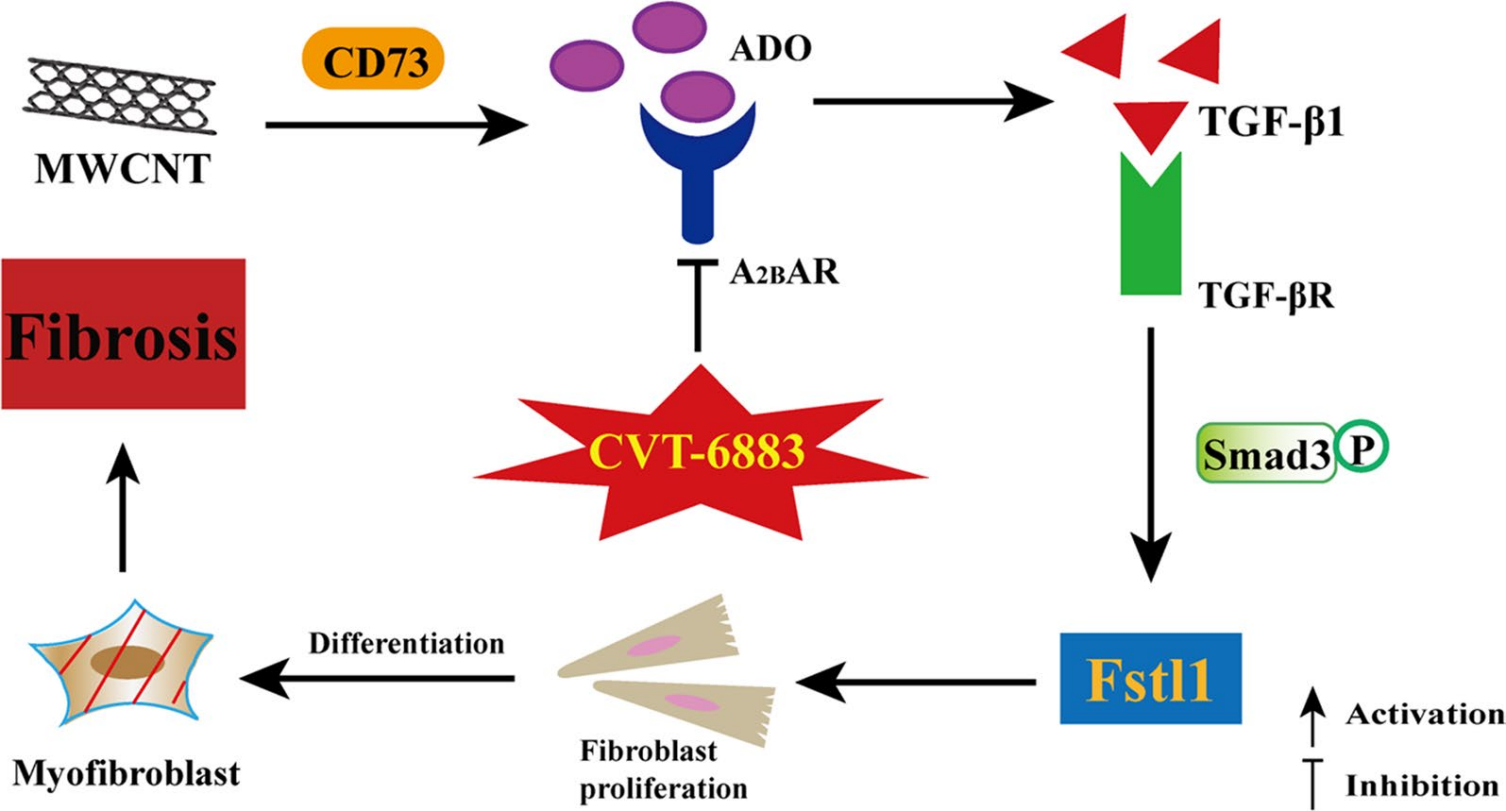
Summary illustration depicting the detrimental role of ADO in MWCNT-induced lung fibrosis. MWCNT activates TGF-β1 signaling-induced fibroblast proliferation and differentiation underlying the production of ADO and engagement of A_2B_AR in the lung

In conclusion, our study identified that excesses extracellular ADO level promoted lung fibrosis following MWCNT exposure, which involved the engagement of the A_2B_AR. Antagonism of A_2B_AR attenuated TGF-β1-induced fibroblast proliferation and differentiation, thereby inhibiting collagen deposition and progressive pulmonary fibrogenesis induced by MWCNT (Fig. 7). To the best of our knowledge, modulation of ADO levels and antagonism of A_2B_AR-mediated responses may be a novel therapeutic approach for MWCNT-induced lung fibrosis.

## Methods

### MWCNT materials and preparation

The MWCNT (TNM7, purity > 98%) has a surface area of > 100 m^2^/g, < 1.5 wt% ash, 0.22 g/cm^3^ tap density, and the nanotube was obtained from Chengdu Organic Chemistry Co., Ltd, Chinese Academy of Sciences (Chengdu, China). Data were provided by the manufacturer. TNM7 is produced by natural gas catalytic decomposition over Ni-based catalyst. The samples were observed using a Hitachi SU 8010 field emission scanning electron microscope (Hitachi, Japan). MWCNT was suspended in dispersion medium (DM) as previously described [47, 48]. DM is Ca^2+^ and Mg^2+^-free phosphate buffered saline (PBS), pH 7.4, supplemented with 5.5 mM d-glucose, 0.6 mg/ml mouse serum albumin (Sigma-Aldrich, St. Louis, MO, USA), and 0.01 mg/ml 1,2-dipalmitoyl-sn-glycero-3-phosphocholine (Sigma-Aldrich).

### Animals

Six to eight weeks male C57BL/6 mice were purchased from Liaoning Changsheng Technology Industrial Co., LTD (Liaoning, China). All experiments involving animals were in accordance with the Ethical Committee for Animal Experiments of Northeast Agricultural University. The mice were housed under environmental conditions (22 ± 2 °C, 55 ± 5% relative humidity) with a 12 h light/dark cycle, and were provided with standard pelleted rodent diet.

### Experimental protocol

A single dose of 50 μl of DM only, or 50 μl of DM containing 40 μg MWCNT was administered by pharyngeal aspiration, which is an alternative to inhalation of administration to deliver a specific dose of an agent into mouse lungs and represents a noninvasive route [49].

Some mice were treatment with CVT-6883 (1 mg/kg, Tocris) in the morning and in the evening (interval was 12 h) for 5 days by intraperitoneal injection. The same formulation and dose of CVT-6883 described above was used in MWCNT studies, where twice daily intraperitoneal injections were given on days 3–7 of the protocol [26, 50].

### Tissue collection and histopathology

The left lobe of the lung was inflated and fixed in 10% neutral buffered formalin for hematoxylin and eosin (H&E) and Masson’s Trichrome. Histological analysis of lung pathology scoring was obtained from stained lung tissue [51]. Fibrotic changes were quantified using the modified Ashcroft scale [49]. The right lung lobes were collected for mRNA and protein analysis.

### Biochemical assays

Blood samples were collected from all animals in EDTA-containing vacutainer tubes. Percentage of neutrophils in peripheral blood of mice were obtained using an automated Auto Hematology Analyzer BC-2600Vet (Mindray, Shenzhen, China).

We also obtained the serum after centrifugated at 3000 × rpm for 10 min at 4 °C. All serum samples were hemolysis-free. Serum LDH activities were measured with a Uni Cel DxC Synchron chemistry system (Beckman Coulter Inc., Fulton, CA, USA).

### Measurement of MPO activity

The lung tissues were homogenized and dissolved in extraction buffer for the analysis of MPO activity [52]. To assess the accumulation of neutrophils in the lung tissues, MPO activities were detected following the respective manufacturer’s instructions (Nanjing Jiancheng Bioengineering Institute, Nanjing, China).

### Bronchoalveolar lavage fluid ADO level

Mice were euthanized for collecting the BALF. Briefly, lungs were lavaged 4 times with 0.3 ml PBS containing 10 μM dipyridamole (Sigma-Aldrich), 10 μM ADA-inhibitor deoxycoformycin (MedChem Express, USA), and 10 μM adenosine 5′-(α,β-methylene) diphosphate (Sigma-Aldrich). The BALF was centrifuged (3000 *g* × 5 min) to remove cells and debris. Twenty microliters of BALF supernatant was analyzed by high-performance liquid chromatography (LC-10AT, Japan) using a 5 µm Hypersil ODS column (250 × 4.6 mm, Dalian Elite Analytical Instruments Co. Ltd, Dalian, China) and a gradient of solutions A (methyl alcohol) and B (0.02 M NH_4_H_2_PO_4_, pH 5.1) at a flow rate of 1 ml/min and run. The ADO peaks were identified and quantified by known external standard curves.

### Analysis of mRNA

Total RNA was isolated from frozen mouse lung tissues using TRIzol reagent (Invitrogen). Real-time quantitative PCR (RT-qPCR) were carried out by a LightCycler 480 II instrument (Roche, Basel, Switzerland) using SYBR Green master mix. Specific oligonucleotide primers used are as follows: CD73, forward: 5′-ATCCGCAAGGAAGAACCC-3′, reverse: 5′-AGTGCCATAGCATCGTAGCC-3′; A_2B_AR, forward: 5′-GAGACTTCCGCTACAGTTTCCA-3′, reverse: 5′-TCATAAGCCCAGACTGAGAGTAGAC-3′; TGF-β1, Sense: 5′-TGAGTGGCTGTCTTTTGACG-3′, Antisense: 5′-TCTCTGTGGAGCTGAAGCAA-3′; Fstl1, forward: 5′-TTATGATGGGCACTGCAAAGAA-3′, reverse: 5′-ACTGCCTTTAGAGAACCAGCC-3′; 18 S ribosomal RNA, forward: 5′-GTAACCCGTTGAACCCCATT-3′, reverse: 5′-CCATCCAATCGGTAGTAGCG-3′. 18 S ribosomal RNA were used as an internal control. Calculations were performed by a comparative 2^−∆∆CT^ method.

### Enzyme-linked immunosorbent assay

The concentration of IL-6 in the BALF was measured by Enzyme-linked immunosorbent assay (ELISA) following the manufacturer’s protocols (R&D Systems, Minneapolis, USA).

### Western blot analysis

Immunoblot analysis was performed as previously described [53, 54] with use of the following antibodies: TGF-β1, collagen I, PDGFR-β, α-SMA, Smad3, p-Smad3 (Cell Signaling Technologies), Fstl1 (R&D Systems), IL-6, HSP47 (Santa Cruz Biotechnology), FSP1 (Sigma-Aldrich), FN1 (Sigma-Aldrich), and HRP-labeled goat anti-mouse IgG or goat anti-rabbit IgG antibody peroxidase-conjugated (ZSGB-BIO, Beijing, China). GAPDH (Hangzhou Goodhere Biotechnology, Hangzhou, China) was used as an internal control for normalization of protein expression.

### Statistical analysis

Results are expressed as mean ± SEM. Differences among groups were evaluated by one-way analysis of variance (ANOVA) followed by Tukey’s post hoc test. A *p*-value < 0.05 was considered as significant. Statistical analyses were carried out using SPSS 19.0 software (SPSS, Chicago, IL, USA).

## Abbreviations

A_2B_AR: A_2B_ adenosine receptor
ADO: adenosine
α-SMA: α-smooth muscle actin
ATP: adenosine triphosphate
BALF: bronchoalveolar lavage fluid
CD39: ectonucleoside triphosphate diphosphohydrolase 1
CD73: ecto-5′-nucleotidase
ECM: extracellular matrix
FN1: fibronectin 1
FSP1: fibroblast-specific protein 1
Fstl1: follistatin-like 1
HSP47: heat shock protein 47
IL-6: interleukin-6
IPF: irreversible pulmonary fibrosis
MPO: myeloperoxidase
MWCNT: multi-walled carbon nanotube
PDGFR-β: platelet-derived growth factor receptor-β
TGF-β1: transforming growth factor-β1
